# Use of Natural Neural Scaffolds Consisting of Engineered Vascular Endothelial Growth Factor Immobilized on Ordered Collagen Fibers Filled in a Collagen Tube for Peripheral Nerve Regeneration in Rats

**DOI:** 10.3390/ijms151018593

**Published:** 2014-10-15

**Authors:** Fukai Ma, Zhifeng Xiao, Danqing Meng, Xianglin Hou, Jianhong Zhu, Jianwu Dai, Ruxiang Xu

**Affiliations:** 1 Fudan University Huashan Hospital, Department of Neurosurgery, National Key Laboratory for Medical Neurobiology, Institutes of Brain Science, Shanghai Medical College-Fudan University, 12 Wulumuqi Zhong Rd., Shanghai 200040, China; E-Mails: kai_008@126.com (F.M.); zhu@fudan.edu.cn (J.Z.); 2 The Affiliated Bayi Brain Hospital, the Military General Hospital of Beijing People’s Liberation Army, No. 5 Nanmen Cang, Dongcheng District, Beijing 100700, China; 3 State Key Laboratory of Molecular Developmental Biology, Institute of Genetics and Developmental Biology, Chinese Academy of Sciences, 3 Nanyitiao, Zhongguancun, Beijing 100190, China; E-Mails: zfxiao@genetics.ac.cn (Z.X.); mengdanqing10@mails.gucas.ac.cn (D.M.); xianglinhou@sohu.com (X.H.)

**Keywords:** engineered vascular endothelial growth factor (VEGF), natural neural scaffolds, biodebradable materials, peripheral nerve regeneration

## Abstract

The search for effective strategies for peripheral nerve regeneration has attracted much attention in recent years. In this study, ordered collagen fibers were used as intraluminal fibers after nerve injury in rats. Vascular endothelial growth factor (VEGF) plays an important role in nerve regeneration, but its very fast initial burst of activity within a short time has largely limited its clinical use. For the stable binding of VEGF to ordered collagen fibers, we fused a collagen-binding domain (CBD) to VEGF through recombinant DNA technology. Then, we filled the ordered collagen fibers-CBD-VEGF targeting delivery system in a collagen tube to construct natural neural scaffolds, which were then used to bridge transected nerve stumps in a rat sciatic nerve transection model. After transplantation, the natural neural scaffolds showed minimal foreign body reactions and good integration into the host tissue. Oriented collagen fibers in the collagen tube could guide regenerating axons in an oriented manner to the distal, degenerating nerve segment, maximizing the chance of target reinnervation. Functional and histological analyses indicated that the recovery of nerve function in the natural neural scaffolds-treated group was superior to the other grafted groups. The guiding of oriented axonal regeneration and effective delivery systems surmounting the otherwise rapid and short-lived diffusion of growth factors in body fluids are two important strategies in promoting peripheral nerve regeneration. The natural neural scaffolds described take advantage of these two aspects and may produce synergistic effects. These properties qualified the artificial nerve conduits as a putative candidate system for the fabrication of peripheral nerve reconstruction devices.

## 1. Introduction

Peripheral nerve injury occurring after trauma may result in poor recovery of function and a subsequent decrease in patient quality of life [1,2]. In contrast to the central nervous system, the peripheral nervous system (PNS) has the ability to regenerate following a nerve lesion [3,4]. However, complex injuries with substantial loss of nerve tissue and a subsequent defect between the nerve ends often require surgical intervention for functional restoration [5]. Currently, the gold standard for restoration of structural and functional nerve regeneration is bridging the defect with an autologous nerve graft, harvested from another site in the body. However, nerve autografts are limited because the process involves sacrifice of one or more healthy nerves, and there may be limited availability of donor tissue [6]. The disadvantages associated with autologous nerve grafts have inspired the search for artificial substitutes that are effective for peripheral nerve reconstruction. Today, tissue engineered nerve grafts composed of a biomaterial and/or growth factors may be the ideal option among the various alternatives [7].

Collagen is the major component of the extracellular matrix (ECM) and widely dispersed in the PNS [8,9]. Collagen is a natural biomaterial that can be used for nerve repair due to its high abundance, low antigenicity, excellent biocompatibility, and biodegradability [10]. In this work, a collagen tube was used to bridge transected nerve stumps and sustain nerve regeneration.

A previous study showed that hollow nerve guidance conduits failed to match the regenerative levels of the autograft and showed poor functional recovery [11]. The inadequate levels of regeneration in a hollow nerve guidance conduit may be attributable to the poor formation of extracellular matrix components during the initial stages of regeneration [12,13].

Thus, in our study, ordered collagen fibers (OCFs) were used in an aligned ECM bridge to facilitate migration of native Schwann cells (SCs) into the site of the lesion and the formation of glial bands of Büngner. The use of regeneration-promoting factors is another strategy for nerve functional recovery.

**Figure 1:**
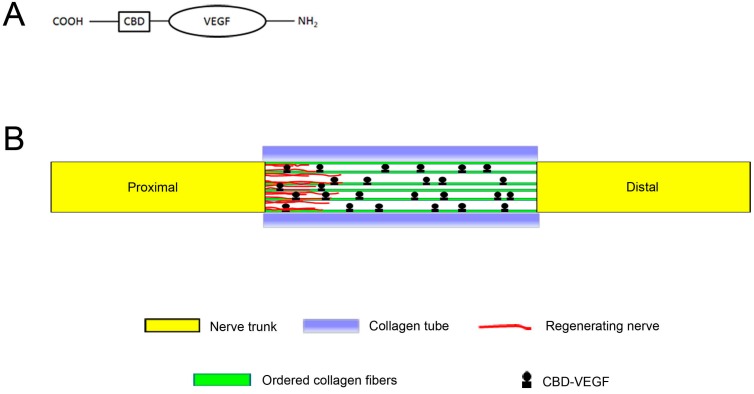
Natural neural scaffold design. (**A**) Diagram of the engineered vascular endothelial growth factor; and (**B**) Schematic diagram showing the natural neural scaffolds for nerve regeneration.

A previous study showed that vascular endothelial growth factor (VEGF) could promote invasion of SCs and neovascularization, which are important steps in nerve regeneration [14]. However, simple delivery of VEGF has no long-term effect because the half-life of VEGF is too short (30–45 min) and metabolic degradation is too fast for sustained activity at the target site for enough time [15]. To increase the immobilization of VEGF on OCFs, we constructed collagen-binding VEGF (CBD-VEGF) by fusing a collagen-binding domain (CBD), consisting of seven amino acids (TKKTLRT), to the *C*-terminal of native VEGF through recombinant DNA technology (Figure 1A). The CBD-VEGF bound stably to the collagen without loss of its biological activity. Then, it was loaded on the OCFs to develop an OCFs-CBD-VEGF targeting delivery system, which was then placed in a collagen tube at a sciatic nerve injury site to promote nerve regeneration.

In this work, the collagen tube filled with the OCFs-CBD-VEGF targeting delivery system was used as a natural neural scaffold to bridge the defect in the rat sciatic nerve (Figure 1B). Regenerative outcomes were evaluated at 6 and 12 weeks after grafting by functional, morphological, and histological assessments.

## 2. Results

### 2.1. General Observation of the Regenerated Nerve

At 12 weeks post-surgery, we found no conspicuous sign of systemic or regional inflammation in rats in the grafted groups. The little immune rejection elicited following implantation in the body was likely because the collagen materials, including the collagen tube and OCFs, are poorly immunogenic. The natural neural scaffolds were replaced by a tissue with nerve-like appearance, which repaired the sciatic nerve defect and joined the stumps.

### 2.2. Motor Functional Assessment of Nerve Regeneration

**Figure 2:**
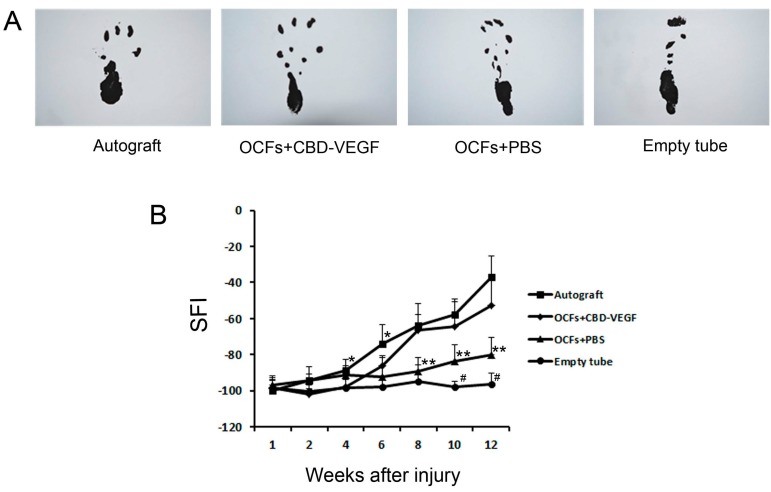
Walking track analysis. (**A**) Photographs of the rat prints on the injured side in the four groups at week 12; and (**B**) The sciatic function index (SFI) values of rats in the three grafted groups and the autograft group. Data are expressed as mean±SD, *n* = 5, ^#^ *p* < 0.05, OCFs + PBS group *vs.* empty tube group; * *p* < 0.05, ** *p* < 0.01, (OCFs + PBS group, autograft group) *vs.* OCFs + CBD-VEGF group.

Walking track analysis was performed to assess the recovery of locomotive function in the rats. The sciatic function index (SFI) values reflect the degree of nerve function. Rats in the four grafted groups showed time-dependent increases in SFI values due to partial functional recovery. However, the SFI value in the empty tube group showed no obvious change. At week 12, the SFI values between the OCFs + CBD-VEGF group and the autograft group were not statistically significantly different (*n* = 5, *p* > 0.05). In contrast to the empty tube group, the OCFs + PBS group showed significantly better SFI levels at weeks 10 and 12 (*n* = 5, *p* < 0.05) (Figure 2).

### 2.3. Sensory Function and Retrograde Tracing Findings

**Figure 3:**
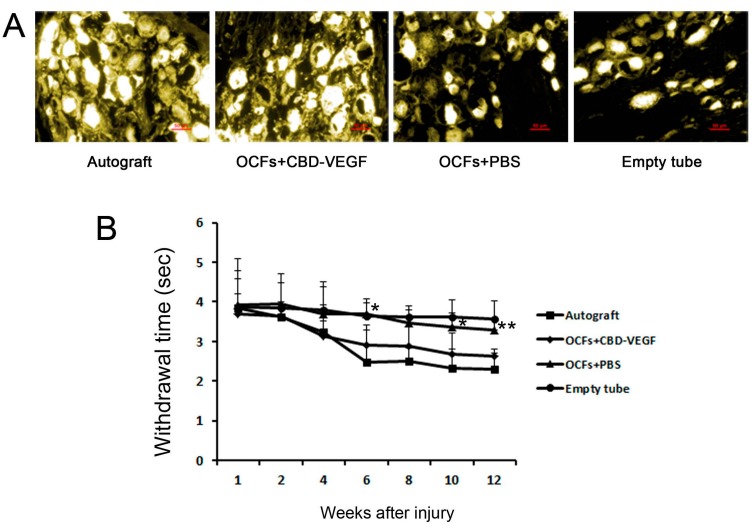
Sensory function and retrograde tracing test. (**A**) Fluorescent micrographs following FG retrograde tracing in the DRGs in the four groups 12 weeks after nerve grafting. *n* = 3. Scale bar, 50 µm; (**B**) Withdrawal time elicited by a hot water stimulus in each group. Data are expressed as mean±SD, *n* = 5, * *p* < 0.05, ** *p* < 0.01, OCFs + CBD-VEGF group *vs.* OCFs + PBS group.

The latency of retraction of the experimental paw from hot water was evaluated. The retraction time in the OCFs + CBD-VEGF group was significantly shorter than the OCFs + PBS group (Figure 3B). In the empty tube group, sensory function did not change significantly with time. There was no significant difference between the OCFs + CBD-VEGF group and the autograft group (*n* = 5, *p* > 0.05). From the results of FG-retrograde tracing performed at 12 weeks, we found FG-labeled neurons in the DRGs in all four groups, indicating that reconnection had occurred between the proximal and distal stumps. The OCFs + CBD-VEGF group showed more nerve fibers connecting the defect than in the OCFs + PBS group (Figure 3A).

### 2.4. Electrophysiological Analysis

**Figure 4:**
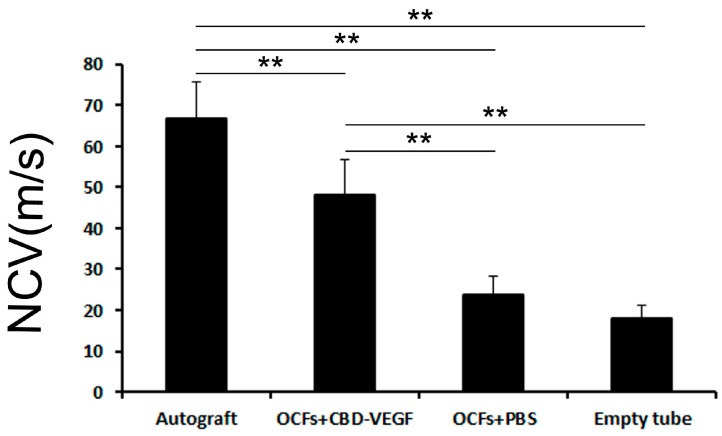
Comparison of the nerve conduction velocity at the injured side in the four groups 12 weeks after nerve grafting.Data are expressed as mean±SD, *n* = 4, ** *p* < 0.01.

An electrophysiological analysis was performed to assess functional reinnervation through the defect. In terms of NCV at 12 weeks after implantation, the value in the autograft group was highest, while it was lowest in the empty tube group. The NCV in the OCFs + CBD-VEGF group was significantly higher than in the OCFs + PBS group (*n* = 4, *p* < 0.01) (Figure 4).

### 2.5. Immunohistochemical Evaluation

**Figure 5:**
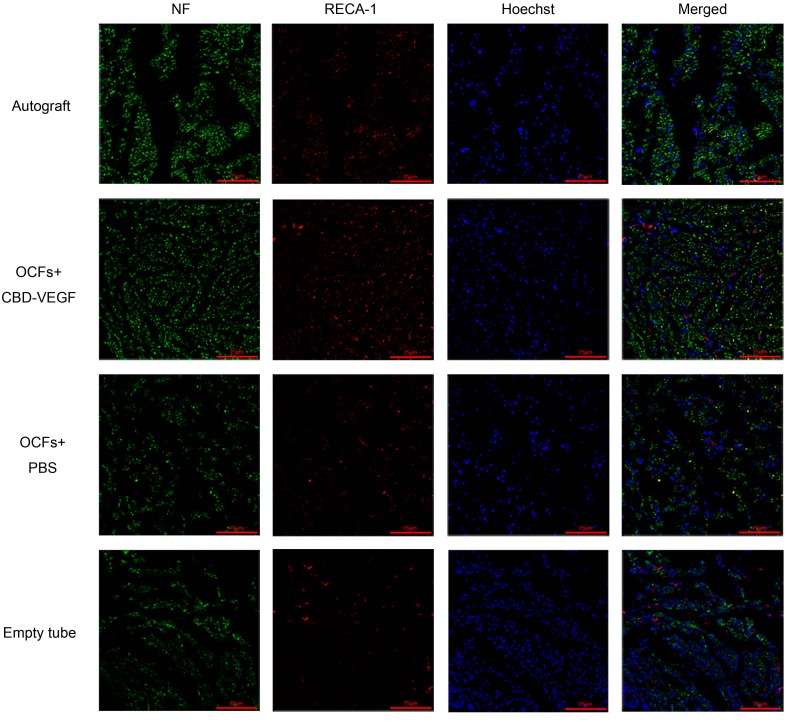
Confocal fluorescent images of transverse sections with anti-NF antibody (green) and anti-RECA-1 antibody (red) at week 12 following implantation for the four groups. Cell nuclei were counterstained with Hoechst dye (blue). Scale bar, 75µm.

**Figure 6:**
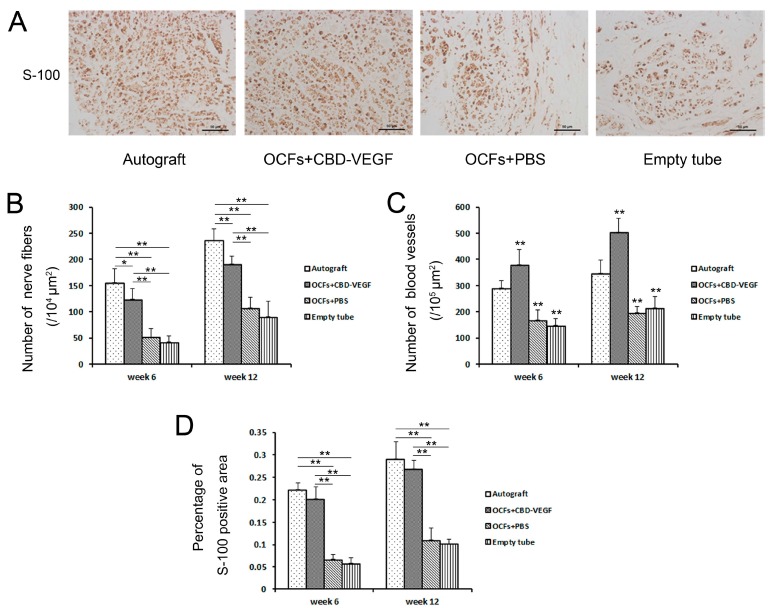
Immunohistochemical evaluation. (**A**) Immunostaining with anti-S-100 antibody. Light micrographs of the regenerating nerve at week 12 after transplantation. Scale bar, 50µm; (**B**) Histogram comparing the number of NF-positive nervefibersamong the four groups; (**C**) Histograms showing the number of RECA-1-positive blood vessels taken from the various groups; (**D**) Statistical analysis of the percentage of S-100-positive area in each group. Data are expressed as mean±SD, *n* = 5, * *p* < 0.05, ** *p* < 0.01 * vs.* autograft group.

At 6 and 12 weeks postimplantation, nerve regeneration behavior was observed under a scanning laser confocal fluorescence microscope (NF for axon fibers, RECA-1 for blood vessels; Figure 5). The OCFs + CBD-VEGF group showed more axonal regeneration (Figure 6B) and capillary vessel formation (Figure 6C) than the OCFs + PBS group (*n* = 5, *p* < 0.01). The blood vessel density in the OCFs + CBD-VEGF group was significantly higher than in the other groups (*n* = 5, *p* < 0.01). Immunostaining images of the Schwann cell marker S-100 were taken with a light microscope (Figure 6A). The percentage of S-100-positive stained area showed no significant difference between the OCFs + CBD-VEGF group and the autograft group (*n* = 5, *p* > 0.05) (Figure 6D).

### 2.6. Histological Assessment

**Figure 7:**
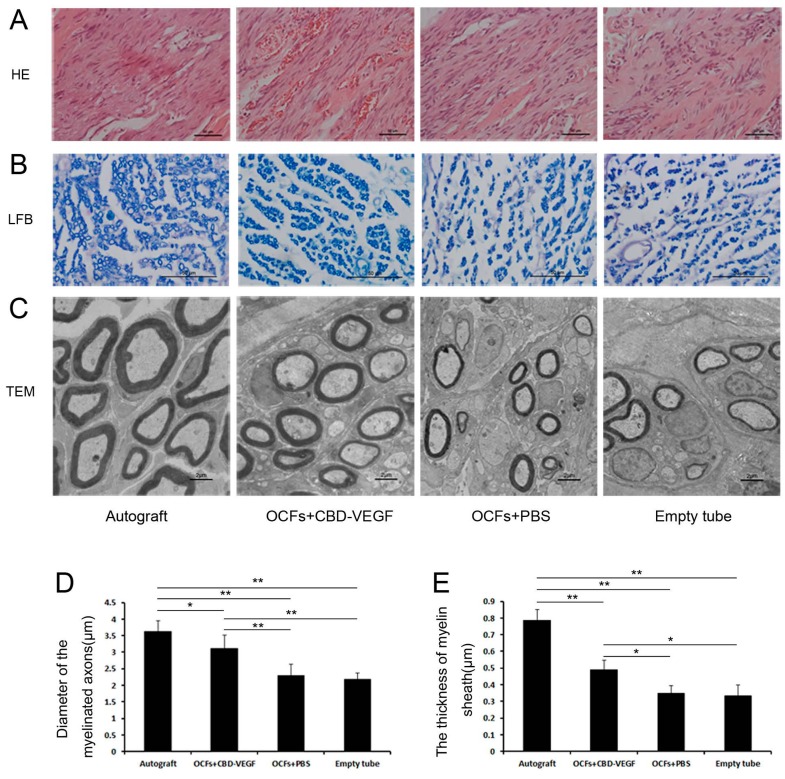
Histological analysis of regenerated nerve at week 12 after injury. (**A**) HE stainingof longitudinal sections in the four groups. Scale bar, 50µm; (**B**) Light micrographs of transverse sections from each group, stained withluxol fast blue (LFB). Scale bar, 50µm; (**C**) Transmission electron microscopy (TEM) images ofultrathin sections in each group. Scale bar, 2µm; (**D**) Statistical analysis of the diameter of myelinated axons, *n* = 5; (**E**) Statistical analysis of myelin sheaththickness, *n* = 3. Data are expressed as mean±SD, * *p* < 0.05, ** *p* < 0.01.

From the results of HE staining, the regenerated nerve in the OCFs + CBD-VEGF group and the OCFs + PBS group showed well-ordered structures compared with the empty tube group (Figure 7A). The group treated with OCFs + CBD-VEGF showed a larger axon diameter (*n* = 5, *p* < 0.01) (Figure 7B,D) and a thicker myelin sheath (*n* = 3, *p* < 0.05) (Figure 7C,E) than those in the other two grafted groups, indicating better nerve regeneration.

### 2.7. Muscle-Mass Ratio and Masson’s Trichrome Staining

**Figure 8:**
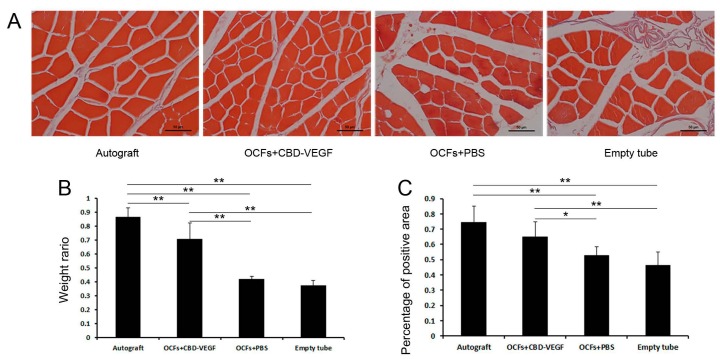
Measurement of gastrocnemius muscle at 12 weeks after surgery. (**A**) Light micrographs of gastrocnemius muscles following Masson’s trichrome staining in each group. Scale bar, 50µm; (**B**) The wet weight ratios of gastrocnemius muscle (injured side/uninjured side) for the threegrafted groups; (**C**) Statistical analysis of the percentage of muscle-positive area in transverse sections from each group. Data are expressed as mean±SD, *n* = 4, * *p* < 0.05, ** *p* < 0.01.

Sciatic nerve injury may result in denervation of target muscles, followed ultimately by muscle atrophy. The wet weight ratio was assessed and Masson’s trichrome staining was performed to evaluate the extent of reinnervation of target muscles at 12 weeks. The OCFs + CBD-VEGF group showed significantly greater wet weight of muscle and larger muscle area compared to the OCFs + PBS group (*n* = 4, *p* < 0.05), indicating better recovery from muscle atrophy (Figure 8). There was no significant difference in the muscle area between the OCFs + CBD-VEGF and autograft groups (*n* = 4, *p* > 0.05).

## 3. Discussion

Ideal tissue engineered nerve grafts have been sought for peripheral nerve repair [16]. Collagen materials were used in our study for bridging extended neural gaps. There was no adverse tissue reaction or serious adhesion at the injury site, likely due to collagen’s excellent biocompatibility. The results in the empty tube group indicated that using a hollow nerve guidance conduit alone for nerve regeneration is not sufficient. Without the formation of an aligned ECM bridge in the nerve guidance conduit, the migration of native SCs into the site of the lesion was limited. This may lead to a reduction in the formation of glial bands of Büngner, essential trophic and topographical guidance structures for regenerating axons [12,17,18,19].

One strategy is the addition of structural intraluminal guidance cues, which may act as a replacement for an unformed or incomplete ECM bridge [20]. In our study, the OCFs, another collagen biomaterial, were used to recapitulate the hierarchical organization and biological function of the native ECM, which is beneficial in promoting nerve regeneration. Despite this, merely providing physical guidance is apparently insufficient, and additional trophic support in the form of biochemical signals is also required [21].

In this study, VEGF was used to activate the collagen materials because of its important role in peripheral nerve regeneration. Delicate control of VEGF protein, in both dosage and localization, is important to enhance its local therapeutic efficacy and decrease possible adverse effects. A previous study showed that CBD could bind specifically to collagen [22]. Thus, CBD was fused to VEGF to use its special binding ability to OCFs. The immobilization of CBD-VEGF on OCFs at the injury site could lead to a high local concentration and prolonged biological effect. This target delivery system (OCFs-CBD-VEGF) was demonstrated to promote nerve regeneration effectively after the injury.

In the current study, the CBD-VEGF in the natural neural scaffolds greatly promoted the outgrowth of Schwann cells and neovascularization following nerve regeneration. Schwann cells are the richest source of neurotrophic factors for peripheral nerve regeneration. Moreover, the newly formed blood vessels in the graft could facilitate the outgrowth of axons by providing sufficient nutrients and oxygen. After functional and histological analyses, the rats treated with CBD-VEGF exhibited the most effective nerve regeneration behavior compared with the other two grafted groups. Moreover, in some analyses, such as the walking track analysis, retraction time, S-100-positive staining area, and muscle-positive staining area, the OCFs + CBD-VEGF group had similar results as the autograft group, with no statistically significant difference between them.

OCFs can guide the direction of nerve regeneration (Figure 7A). According to a previous study, oriented regrowth guidance played an important role in nerve regeneration and functional recovery [23]. After injury, the regenerated nerve fibers may disperse in the nerve guidance conduit. Misdirected regrowth may result in inappropriate target reinnervation and dysfunctional connections [24,25,26]. It was demonstrated by our results that OCFs could provide the regenerating nerve with correct linear order guidance for growth, which may contribute to the functional recovery in the OCFs-treated group compared with the empty tube group (Figure 2B).

The use of CBD-VEGF may be practical for clinical use. The application of free VEGF to nerve injuries has no long-term effect on nerve regeneration because of the rapid diffusion and short half-life time in body fluids [27,28]. To sustain an effective VEGF concentration at the injury site, high doses or periodic injections are required, which become expensive and/or impractical. Furthermore, high doses of VEGF can lead to the formation of hemangiomas, and the diffusion of VEGF may cause undesirable side effects elsewhere [29]. Transplanting genetically modified cells that overexpress VEGF to accelerate tissue healing may result in immunological rejection [30,31]. Using an adeno-associated viral VEGF gene may present safety problems [32,33]. Use of a mini-osmotic pump to deliver VEGF may cause infection [34]. Some materials have low affinities for VEGF and the covalent conjugation that has been used to immobilize VEGF on materials may be harmful to the human body [35]. The strategy used here to deliver VEGF required no toxic reagents and enables VEGF conjugation via mild reactions without loss of its biological activity. This is in contrast to covalent conjugation through conventional condensation reactions.

## 4. Material and Methods

### 4.1. Preparation of OCFs and Collagen Tubes

**Figure 9:**
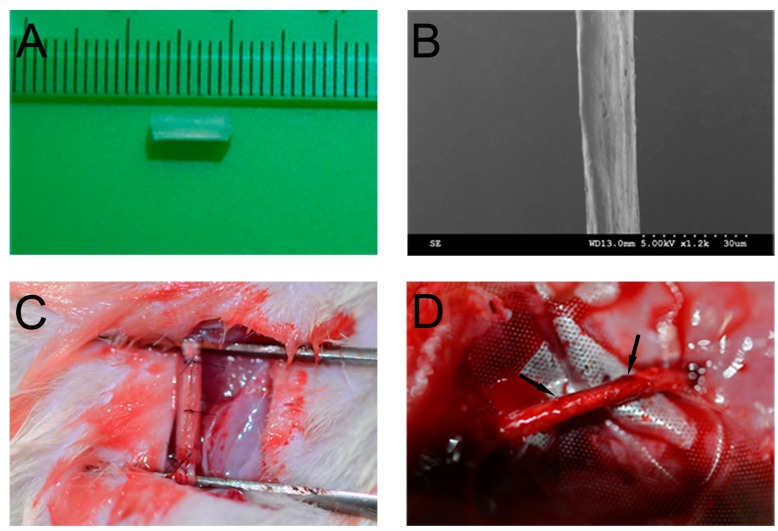
The biomaterials, surgery, and regenerated nerve. (**A**) Photograph of the collagen tube; (**B**) SEM image of ordered collagen fibers; (**C**) The sciatic nerve was transected and bridged by the natural neural scaffolds; (**D**) Photograph of the regenerated nerve at 12 weeks post-implantation. The arrow indicates the two ends of the regenerated nerve.

OCFs were prepared from bovine aponeuroses according to our previous report, with minor modifications [36]. Fresh white aponeuroses were separated from muscles and cleaned with cold distilled water. Residual muscle, connective tissue, fat, and cellular components were removed with 1% tri(*n*-butyl) phosphate (TnBP; Aldrich, Munich, Germany) in 50 mM Tris–HCl buffer (pH 8.0) for 48 h at 4 °C. After removing the residual agents, OCFs were separated from the processed aponeurosis followed by freeze-drying. The collagen tube was fabricated from collagen membrane (Zhenghai Biotechnology, Shandong, China). Briefly, after immersion in distilled water, the collagen membrane was rolled on a cylindrical mold. Then, collagen solution was evenly spread on the rolled collagen membrane. After air-drying, the collagen tube was treated with cross-linking solution (30 mM 1-ethyl-3-(3-dimethyllaminopropyl)carbodiimide hydrochloride (EDC) and 10 mM *N*-hydroxysuccinimide (NHS) in 50 mM Fatty Acid Methyl Ester Sulfonate (MES) solution, pH 5.5) for 12 h. After washing with NaH_2_PO_4_ (0.1 M) and distilled water, the collagen tube was removed from the mold, followed by freeze-drying. OCFs and the collagen tube (Figure 9A) were sterilized by ^60^Co irradiation before use. The surface morphology of OCFs was analyzed by scanning electron microscopy (SEM; Hitachi, Tokyo, Japan; Figure 9B).

### 4.2. Production of CBD-VEGF

CBD-VEGF was prepared as described previously [37]. The gene for CBD-VEGF was generated by polymerase chain reaction (PCR), and then inserted into plasmid pET-28a. After BL21(DE3)-strain *Escherichia coli* were transformed with the target vector, protein expression was induced with 1 mM isopropyl β-D-thiogalactopyranoside (IPTG) for 5 h. Then, the protein was purified by nickel chelate chromatography with HiTrap heparin HP columns (GE Healthcare, Chalfont St Giles, UK). Finally, 0.1 nM protein was loaded on a bundle of OCFs for 30 min before transplantation.

### 4.3. Animals and Surgical Procedure

All experimental procedures were performed according to the guide for the care and use of laboratory animals from National Institutes of Health and approved by the Animal Experimental Committee of Fudan University (Shanghai, China).

Adult female Sprague–Dawley rats, weighing 200–220 g, were used to evaluate nerve regeneration performance. The animals were randomized into three grafted groups and an autograft group (*n* = 21 each). Animals were anesthetized by intraperitoneal injection of sodium pentobarbital (40 mg/kg body weight) before the sciatic nerve was exposed by making a skin incision and splitting the underlying muscles in the right lateral thigh. The nerve segment was removed and replaced by a 8-mm-long collagen tube. After that, the sciatic nerve defect was bridged by: (1) an empty collagen tube; (2) a collagen tube + OCFs + PBS; or (3) a collagen tube + OCFs + CBD-VEGF (Figure 2C). In the autograft group, the removed sciatic nerve segment was rotated 180° and sutured to both nerve stumps. After surgery, animals in all groups were housed in their cages and fed routinely under normal conditions.

### 4.4. Functional Assessment of Nerve Regeneration

Walking track analysis was performed in all animals every other week. The sciatic function index (SFI) value was calculated using the formula proposed by Bain *et al*. [38]. The paw length (PL), toe spread (TS), and intermediary toe spread (ITS) were collected on both the normal (N) and the experimental (E) hind legs.

SFI = −38.3 × (EPL − NPL)/NPL + 109.5 × (ETS − NTS)/NTS + 13.3 × (EITS − NITS)/NITS − 8.8
(1)

The SFI varies from 0 to −100: scores around 0 indicate normal nerve function and around −100 indicates complete loss of function.

### 4.5. Assessment of Sensory Recovery

To evaluate the recovery of sensory nerve function, we measured the foot withdrawal reflex elicited by a hot water stimulus every other week [39,40]. Briefly, the plantar surface of the right paw was immersed in a 50 °C water bath until the rat first lifted the hindpaw. If the rat did not retract within 5 s, its hind paw was removed from the water to prevent tissue damage. The time interval was recorded by an investigator blinded to the group assignments.

### 4.6. Fluorogold Retrograde Tracing

Retrograde tracing with the retrograde fluorescent tracer Fluorogold (FG; Fluorochrome, Denver, CO, USA) was used to examine the reinnervation between proximal and distal stumps. At 10 weeks post-transplantation, after rats were anesthetized, the surgical site was re-opened. Then, 3 μL of 5% (5 mg/100 μL, dissolved in saline) Fluorogold were injected at the site 4 mm from the distal transplant-sciatic nerve interface. Animals were sacrificed 2 weeks after injection, and the dorsal root ganglia (DRG) at L4–L6 were dissected. The specimens were frozen immediately at −80 °C and cut into transverse 10-µm-thick sections on a freezing microtome. The sections were mounted on positively charged slides followed by observation under a fluorescence microscope (Carl Zeiss, Jena, Germany).

### 4.7. Electrophysiological Assessment

Electrophysiological tests were carried out on rats 12 weeks after surgery. Under anesthesia, the injury site was carefully re-exposed and dissected from the surrounding tissues. A stimulating electrode was placed in the proximal end of the nerve trunk, while two recording needle electrodes were placed in the proximal and distal end of the nerve trunk. A ground electrode was placed in the surrounding muscle tissues to remove conduction of stimulation through muscle tissues. Nerve conduction velocity (NCV) was recorded using an electromyography system (RM6240, Chengdu, China).

### 4.8. Immunohistochemical Evaluation

Animals were sacrificed at 6 and 12 weeks for immunohistochemical analyses. The distal ends of regenerated nerves were dissected carefully, post-fixed overnight at 4 °C, transferred to 20% sucrose (3 days at 4 °C) and then 30% sucrose (3 days at 4 °C). After snap-freezing in liquid nitrogen, they were cut into transverse sections 10 µm thick. The specimens were stained with primary antibodies against neurofilament-200 (NF, 1:500 dilution, Abcam, Cambridge, MA, USA) and rat endothelial cell cytoplasmic antigen (RECA-1, 1:100 dilution, Serotec, Kidlington, UK). Detection was accomplished by incubation with Alexa 488-conjugated donkey anti-rabbit secondary antibody (Invitrogen, Grand Island, NY, USA) and Alexa 594-conjugated donkey anti-mouse secondary antibody (Invitrogen) for 1 h. Hoechst 33342 was used for nuclear staining. The stained sections were observed under a scanning laser confocal fluorescence microscope (Leica, Wetzlar, Germany).

Specimens for anti-S-100 staining were embedded in paraffin wax and then cut into 5-μm transverse sections. After antigen retrieval, the sections were incubated with mouse anti-S-100 antibody (1:100 dilution, Zeta, Sierra Madre, CA, USA) overnight at 4 °C. After incubation with secondary antibody (goat anti-mouse IgG, Invitrogen) and then with HRP-streptavidin (Jackson Immunoresearch, West Grove, PA, USA), the stained sections were observed under a light microscope (Nikon, Tokyo, Japan). The percentage of S-100 (positive staining area/total area) in each photograph was calculated using the Image-Pro Plus software (Media Cybernetics, Rockville, MD, USA).

### 4.9. Histological Evaluation

At week 12, the regenerated nerves were fixed in 4% (*v*/*v*) formaldehyde for 48 h and then embedded in paraffin wax and cut into 5-μm-thick longitudinal sections that were then stained with hematoxylin and eosin (HE) to detect structures.

Luxol fast blue (LFB) staining was conducted for the myelin sheath at week 12. The transverse section was placed in 95% alcohol and stained in LFB solution (Sigma, St. Louis, MO, USA) overnight at 37 °C. After differentiation in lithium carbonate solution and 70% ethyl alcohol, the slide was counterstained with cresyl violet. Finally, the slide was mounted with resinous medium and examined under a light microscope (Nikon). The diameters of the myelinated axons in each photograph were evaluated using the Image-Pro Plus software.

For transmission electron microscopy (TEM) analysis, the distal end of regenerated nerve at week 12 was cut into transverse ultrathin sections of 50–60 nm thick to observe myelin sheath regeneration. The sections were stained with lead citrate and uranyl acetate and then examined by TEM (H-7650B, Hitachi, Tokyo, Japan). The thickness of myelin sheaths in each photograph was evaluated using the Image-Pro Plus software.

### 4.10. Muscle-Mass Ratio and Masson’s Trichrome Staining

At 12 weeks after implantation, the gastrocnemius muscles of the implanted (left) and contralateral normal (right) sides were dissected. The muscle-mass ratio (wet weight of right side/left side) was measured to evaluate the muscular atrophy caused by sciatic nerve denervation. Then, the midbellies of the muscle samples were embedded in paraffin wax and cut into 5-μm-thick transverse sections for Masson’s trichrome staining. The percentage of muscle fibers (positive staining area/total area) was calculated using the Image-Pro Plus software.

### 4.11. Statistical Analysis

Data are presented as mean ± standard deviations (SD). Statistical analysis was performed with one-way Analysis of Variance (ANOVA) followed by the Student-Newman-Keuls (SNK) *post hoc* test using the SPSS software (version 13.0, SPSS Inc., Chicago, IL, USA). Differences were considered statistically significant at *p* < 0.05 (indicated as * *p* < 0.05 or ** *p* < 0.01).

## 5. Conclusions

In the present study, natural neural scaffolds constructed from collagen tubes, filled with the OCFs-CBD-VEGF targeting delivery system, were used to reconstruct nerve defects in adult rat sciatic nerves. As can be seen from the findings, the natural neural scaffolds achieved satisfying nerve regeneration outcomes associated with morphological and functional improvements that were similar to those of the autograft. The natural neural scaffolds described may be an effective therapeutic technique for peripheral nerve regeneration.

## References

[1] Kemp S.W., Walsh S.K., Midha R. (2008). Growth factor and stem cell enhanced conduits in peripheral nerve regeneration and repair. Neurol. Res..

[2] Huang J., Zhang Y., Lu L., Hu X., Luo Z. (2013). Electrical stimulation accelerates nerve regeneration and functional recovery in delayed peripheral nerve injury in rats. Eur. J. Neurosci..

[3] Gu X., Ding F., Yang Y., Liu J. (2011). Construction of tissue engineered nerve grafts and their application in peripheral nerve regeneration. Prog. Neurobiol..

[4] English A.W., Cucoranu D., Mulligan A., Rodriguez J.A., Sabatier M.J. (2011). Neurotrophin-4/5 is implicated in the enhancement of axon regeneration produced by treadmill training following peripheral nerve injury. Eur. J. Neurosci..

[5] Campbell W.W. (2008). Evaluation and management of peripheral nerve injury. Clin. Neurophysiol..

[6] Lundborg G., Richard P. (2003). Bunge memorial lecture. Nerve injury and repair—A challenge to the plastic brain. JPNS.

[7] Chalfoun C.T., Wirth G.A., Evans G.R. (2006). Tissue engineered nerve constructs: Where do we stand?. J. Cell. Mol. Med..

[8] Yurchenco P.D., Smirnov S., Mathus T. (2002). Analysis of basement membrane self-assembly and cellular interactions with native and recombinant glycoproteins. Methods Cell Biol..

[9] Longo F.M., Hayman E.G., Davis G.E., Ruoslahti E., Engvall E., Manthorpe M., Varon S. (1984). Neurite-promoting factors and extracellular matrix components accumulating *in vivo* within nerve regeneration chambers. Brain Res..

[10] Li X., Feng Q., Liu X., Dong W., Cui F. (2006). Collagen-based implants reinforced by chitin fibres in a goat shank bone defect model. Biomaterials.

[11] Ribeiro-Resende V.T., Koenig B., Nichterwitz S., Oberhoffner S., Schlosshauer B. (2009). Strategies for inducing the formation of bands of Bungner in peripheral nerve regeneration. Biomaterials.

[12] Koh H.S., Yong T., Teo W.E., Chan C.K., Puhaindran M.E., Tan T.C., Lim A., Lim B.H., Ramakrishna S. (2010). *In vivo* study of novel nanofibrous intra-luminal guidance channels to promote nerve regeneration. J. Neural Eng..

[13] Chew S.Y., Mi R., Hoke A., Leong K.W. (2007). Aligned protein-polymer composite fibers enhance nerve regeneration: A potential tissue-engineering platform. Adv. Funct. Mater..

[14] Sondell M., Lundborg G., Kanje M. (1999). Vascular endothelial growth factor stimulates Schwann cell invasion and neovascularization of acellular nerve grafts. Brain Res..

[15] Frey S.P., Jansen H., Raschke M.J., Meffert R.H., Ochman S. (2012). VEGF improves skeletal muscle regeneration after acute trauma and reconstruction of the limb in a rabbit model. Clin. Orthop. Relat. Res..

[16] Tan A., Rajadas J., Seifalian A.M. (2012). Biochemical engineering nerve conduits using peptide amphiphiles. J. Control. Release.

[17] Mukhatyar V., Karumbaiah L., Yeh J., Bellamkonda R. (2009). Tissue engineering strategies designed to realize the endogenous regenerative potential of peripheral nerves. Adv. Mater..

[18] Belkas J.S., Shoichet M.S., Midha R. (2004). Peripheral nerve regeneration through guidance tubes. Neurol. Res..

[19] Hoffman-Kim D., Mitchel J.A., Bellamkonda R.V. (2010). Topography, cell response, and nerve regeneratio. Annu. Rev. Biomed. Eng..

[20] Bellamkonda R.V. (2006). Peripheral nerve regeneration: An opinion on channels, scaffolds and anisotropy. Biomaterials.

[21] Pereira Lopes F.R., Frattini F., Marques S.A., Almeida F.M., de Moura Campos L.C., Langone F., Lora S., Borojevic R., Martinez A.M. (2010). Transplantation of bone-marrow-derived cells into a nerve guide resulted in transdifferentiation into Schwann cells and effective regeneration of transected mouse sciatic nerve. Micron.

[22] De Souza S.J., Brentani R. (1992). Collagen binding site in collagenase can be determined using the concept of sense-antisense peptide interactions. J. Biol. Chem..

[23] Hu X., Huang J., Ye Z., Xia L., Li M., Lv B., Shen X., Luo Z. (2009). A novel scaffold with longitudinally oriented microchannels promotes peripheral nerve regeneration. Tissue Eng. Part A.

[24] Brushart T.M., Mathur V., Sood R., Koschorke G.M. (1995). Dispersion of regenerating axons across enclosed neural gaps. J. Hand Surg..

[25] Madison R.D., Archibald S.J., Lacin R., Krarup C. (1999). Factors contributing to preferential motor reinnervation in the primate peripheral nervous system. J. Neurosci..

[26] Hamilton S.K., Hinkle M.L., Nicolini J., Rambo L.N., Rexwinkle A.M., Rose S.J., Sabatier M.J., Backus D., English A.W. (2011). Misdirection of regenerating axons and functional recovery following sciatic nerve injury in rats. J. Comp. Neurol..

[27] Silva E.A., Mooney D.J. (2007). Spatiotemporal control of vascular endothelial growth factor delivery from injectable hydrogels enhances angiogenesis. J. Thromb. Haemost..

[28] Eppler S.M., Combs D.L., Henry T.D., Lopez J.J., Ellis S.G., Yi J.H., Annex B.H., McCluskey E.R., Zioncheck T.F. (2002). A target-mediated model to describe the pharmacokinetics and hemodynamic effects of recombinant human vascular endothelial growth factor in humans. Clin. Pharmacol. Ther..

[29] Lee R.J., Springer M.L., Blanco-Bose W.E., Shaw R., Ursell P.C., Blau H.M. (2000). VEGF gene delivery to myocardium: Deleterious effects of unregulated expression. Circulation.

[30] Liu G., Sun X., Bian J., Wu R., Guan X., Ouyang B., Huang Y., Xiao H., Luo D., Atala A. (2013). Correction of diabetic erectile dysfunction with adipose derived stem cells modified with the vascular endothelial growth factor gene in a rodent diabetic model. PLoS One.

[31] Nauta A., Seidel C., Deveza L., Montoro D., Grova M., Ko S.H., Hyun J., Gurtner G.C., Longaker M.T., Yang F. (2013). Adipose-derived stromal cells overexpressing vascular endothelial growth factor accelerate mouse excisional wound healing. Mol. Ther..

[32] Saeed M., Saloner D., Martin A., Do L., Weber O., Ursell P.C., Jacquier A., Lee R., Higgins C.B. (2007). Adeno-associated viral vector-encoding vascular endothelial growth factor gene: Effect on cardiovascular MR perfusion and infarct resorption measurements in swine. Radiology.

[33] Galeano M., Deodato B., Altavilla D., Cucinotta D., Arsic N., Marini H., Torre V., Giacca M., Squadrito F. (2003). Adeno-associated viral vector-mediated human vascular endothelial growth factor gene transfer stimulates angiogenesis and wound healing in the genetically diabetic mouse. Diabetologia.

[34] Boodhwani M., Mieno S., Voisine P., Feng J., Sodha N., Li J., Sellke F.W. (2006). High-dose atorvastatin is associated with impaired myocardial angiogenesis in response to vascular endothelial growth factor in hypercholesterolemic swine. J. Thorac. Cardiovasc. Surg..

[35] Davies N.H., Schmidt C., Bezuidenhout D., Zilla P. (2012). Sustaining neovascularization of a scaffold through staged release of vascular endothelial growth factor-A and platelet-derived growth factor-BB. Tissue Eng. Part A.

[36] Lin H., Chen B., Wang B., Zhao Y., Sun W., Dai J. (2006). Novel nerve guidance material prepared from bovine aponeurosis. J. Biomed. Mater. Res. Part A.

[37] Zhang J., Ding L., Zhao Y., Sun W., Chen B., Lin H., Wang X., Zhang L., Xu B., Dai J. (2009). Collagen-targeting vascular endothelial growth factor improves cardiac performance after myocardial infarction. Circulation.

[38] Bain J.R., Mackinnon S.E., Hunter D.A. (1989). Functional evaluation of complete sciatic, peroneal, and posterior tibial nerve lesions in the rat. Plast. Reconstr. Surg..

[39] Derby A., Engleman V.W., Frierdich G.E., Neises G., Rapp S.R., Roufa D.G. (1993). Nerve growth factor facilitates regeneration across nerve gaps: Morphological and behavioral studies in rat sciatic nerve. Exp. Neurol..

[40] Young C., Miller E., Nicklous D.M., Hoffman J.R. (2001). Nerve growth factor and neurotrophin-3 affect functional recovery following peripheral nerve injury differently. Restor. Neurol. Neurosci..

